# Association between pulmonary function tests and lipid levels

**DOI:** 10.1097/MD.0000000000045111

**Published:** 2025-10-10

**Authors:** Kadriye Akpinar, Alev Lazoğlu Özkaya

**Affiliations:** a Biochemistry Laboratory, Ministry of Health, Burdur State Hospital, Burdur, Türkiye; b Occupational Medicine Physician of Provincial Health Directorate, Burdur, Türkiye; c Department of Biochemistry, Erzurum Regional Training and Research Hospital, Erzurum, Türkiye.

**Keywords:** cholesterol, HDL, hyperlipidemia, monocyte, pulmonary function tests

## Abstract

This study aimed to investigate the association between pulmonary function tests (PFTs) and biochemical parameters, including lipid levels, monocyte-to-high-density lipoprotein (HDL) ratio (MHR), glucose, and glycated hemoglobin (HbA1c), undergoing routine health checkups. Cholesterol, low-density lipoprotein cholesterol, HDL cholesterol, triglyceride (TG), and glucose levels of a total of 153 subjects (51% male) were analyzed using the Abbott Architect c8000 auto-analyzer. HbA1c was measured using the Lifotronic H9 HPLC system. Monocyte count was determined using the Abbott CELL-DYN Ruby fully automated blood counter. PFTs were conducted using a spirometer, and data were analyzed using SPSS version 27.0. Glucose, cholesterol, low-density lipoprotein cholesterol, HDL cholesterol, TG, and HbA1c levels were significantly higher in the overweight and obese group than in the control group (*P* < .05). Similarly, glucose, cholesterol, TG, and HbA1c levels were significantly higher in the diabetic and prediabetic groups compared to the control group (*P* < .05). The high lipid group exhibited elevated monocyte counts and MHR, along with reduced HDL levels compared to the optimal lipid group. Smokers had a significantly higher MHR in smokers (0.012) than nonsmokers (0.010), and males (0.013) exhibited higher MHR values than females (0.008). Significant differences in PFTs were observed for smokers, who exhibited lower forced vital capacity (FVC) (3.70 ± 0.99 L) and forced expiratory volume in 1 second (FEV1) (3.05 ± 0.77 L/s) compared to nonsmokers (FVC: 4.23 ± 1.16 L and FEV1: 3.44 ± 0.84 L/s). Similarly, females had lower FVC (3.14 ± 0.59 L), FEV1 (3.05 ± 0.77 L/s), and peak expiratory flow (5.1 ± 1.16 L/s) compared to males (FVC: 4.23 ± 1.16 L, FEV1: 3.44 ± 0.84 L/s, peak expiratory flow: 7.3 ± 1.89 L/s). In the multivariable regression model, the associations of lipids, body mass index, and smoking were not independently significant, whereas age and sex emerged as the strongest factors associated with pulmonary function. Older age was linked to significant declines in FVC and FEV1, and males exhibited significantly higher lung volumes than females. In conclusion, age and sex are significantly associated with pulmonary function, while the associations of smoking, body mass index, HbA1c, and lipid levels appear to be more complex and require further investigation.

## 1. Introduction

Hyperlipidemia (HL) is a condition characterized by elevated lipid levels in the body, which may arise from genetic predispositions or acquired disorders.^[1]^ It specifically refers to elevated levels of total cholesterol (TC), low-density lipoprotein cholesterol (LDL-C), very low-density lipoprotein cholesterol, and triglycerides (TGs) compared to the general population.^[2]^ HL is classified into 2 types: primary and secondary. Primary HL is inherited and often runs in families, whereas secondary HL typically develops later in life due to an unhealthy diet, certain medications (e.g., amiodarone, glucocorticoids), or underlying hormonal and metabolic disorders such as hypothyroidism and uncontrolled diabetes.^[3]^ The rising prevalence of obesity and type 2 diabetes mellitus (DM) has contributed to an increased incidence of HL.^[4]^ HL is a common condition worldwide and is considered one of the major preventable risk factors, along with hypertension and smoking, for the development of atherosclerotic cardiovascular disease (CVD). CVD remains the leading cause of morbidity and mortality worldwide, posing significant health challenges and economic burdens. Studies have shown that measuring serum lipid concentrations is one of the most effective methods for assessing CVD risk.^[5]^ Although the presence of chronic diseases is a known risk factor for HL, it can also occur in individuals without chronic conditions, including those in the pediatric population. For this reason, early lipid screening is recommended to help prevent future comorbidities such as metabolic syndrome, obesity, DM, and CVD. The frequency of lipid screening is determined by factors such as the patient’s age, sex, and the presence of additional risk factors. As part of a global risk assessment, lipid screening is recommended every 5 years for young adults, and every 2 years for men over 40 years of age and women over 50 years (or postmenopause). Screening should be performed at least annually in individuals with DM, atherosclerotic CVD, chronic kidney disease, or those presenting risk factors such as smoking, obesity, hypertension, dyslipidemia, or a family history of CVD in first-degree relatives.^[5,6]^

The lung is a structurally specialized organ of the human body and maintains a unique relationship with cholesterol.^[7]^ Cholesterol overload in various subcellular compartments of macrophages may stimulate stress-induced cytokine production in the endoplasmic reticulum, enhance pro-inflammatory signaling via Toll-like receptors, trigger inflammatory activation, or suppress pro-inflammatory gene expression. Alterations in cholesterol synthesis also play a role in the antiviral response of macrophages and in the proliferation of T cells. The abnormal buildup of lipid-laden macrophage “foam cells” in the lungs is implicated in numerous lung diseases. However, the underlying mechanism remains unclear. Recent studies have identified impaired lipid metabolism as a contributing factor in the progression of various lung diseases, including chronic obstructive pulmonary disease (COPD), asthma, acute respiratory distress syndrome, lung cancer, pulmonary arterial hypertension, and lung fibrosis.^[7–9]^ Spirometry is a noninvasive, simple, cost-effective, reproducible, and standardized method for measuring pulmonary function tests (PFTs). PFTs provide valuable information for the diagnosis, screening, treatment, and follow-up of patients with respiratory disorders. They enable assessment of the large and small airways, lung parenchyma, and the size and integrity of the pulmonary capillary bed. Although they do not provide a definitive diagnosis on their own, they aid in the diagnosis of various respiratory diseases.^[10]^ Approximately 3% to 8% of leukocytes in peripheral blood are monocytes. Lipid-laden macrophages form as a result of monocyte activation, which leads to the synthesis and release of pro-inflammatory and pro-oxidant cytokines. They play a role in regulating inflammatory processes. High-density lipoprotein (HDL) cholesterol, on the other hand, protects the endothelium from LDL cholesterol, prevents LDL oxidation, and exerts antithrombotic, anti-inflammatory, and antioxidant effects.^[11]^ The monocyte-to-HDL ratio (MHR) is a novel, easily calculable marker of inflammation and oxidative stress, and serves as a practical indicator for the presence and prognosis of diseases related to these processes.

Few studies have explored the relationship between PFTs assessed via spirometry and serum levels of lipids, glucose, or glycated hemoglobin (HbA1c).^[12,13]^ This study aimed to clarify the relationships between PFTs and lipid levels, as well as MHR, fasting glucose (FG), and HbA1c levels, in individuals undergoing health checkups in Turkey, from the perspective of preventive medicine.

## 2. Materials and methods

### 2.1. Study design

A total of 153 individuals (78 males and 75 females), aged over 18 years (mean age ± standard deviation: 42 ± 10; minimum–maximum age:19–69 years), were included in the study using a consecutive sampling method. These participants applied for routine occupational health checkups at the Occupational Health and Safety Center as part of an annual screening program and underwent PFT between January 2020 and December 2022. Exclusion criteria included chronic use of corticosteroids, pregnancy, malignancy, infection, chronic lung disease, connective tissue disease, and thyroid disorders in all subjects. These exclusion criteria were determined based on participants’ self-reported medical history collected during the health checkup interview.

Height, weight, body mass index (BMI = weight [kg]/height [m ]^2^), and medical history, including CVD, diabetes, cigarette smoking, and alcohol use, were recorded for all subjects. Fasting venous blood samples were collected from individuals in the morning after 8 to 12 hours of fasting. Samples for biochemical analysis were drawn into gel vacuum tubes (Vacusera, Türkiye), while whole blood samples for HbA1c and monocyte count were collected in ethylenediaminetetraacetic acid tubes (Vacusera, Türkiye). FG, TC, TG, HDL cholesterol (HDL-C), and LDL-C levels were measured using the Architect c8000 auto-analyzer (Abbott, Abbott Park) with enzymatic photometric methods (glucose oxidase method for FG). LDL cholesterol was calculated using the Friedewald equation when TG levels were below 400 mg/dL, according to the formula: LDL = TC − [HDL + (TG/5)]. HbA1c was analyzed using the Lifotronic H9 (Lifotronic Technology Co., Ltd, Shenzhen, China) via ion-exchange high-performance liquid chromatography, following the guidelines of the National Glycohemoglobin Standardization Program.^[14]^ Monocyte count was measured using the CELL-DYN Ruby fully automated hematology analyzer (Abbott, Abbott Park), which performs a 3-dimensional optical complete blood count with a 5-part white blood cell differential analysis. MHR was calculated as the ratio of the absolute monocyte count (×10^3^/μL) to the HDL-C level (mg/dL).^[15]^

PFTs, including forced vital capacity (FVC), forced expiratory volume in 1 second (FEV1, L/s), percent predicted FEV1 (%) and FVC (%), FEV1/FVC ratio (%), and peak expiratory flow (PEF, L/s), were performed using conventional spirometry with the MIR MiniSpir PC-Based USB Spirometer (MIR Medical International Research, Waukesha), in accordance with the recommendations of the American Thoracic Society.^[16]^ According to the Global Initiative for Chronic Obstructive Lung Disease, FEV1 < 80%, FVC < 80%, and FEV1/FVC < 70% indicate airflow limitation and suggest the possibility of COPD.^[17]^

In this study, subjects were categorized into subgroups based on their BMI, lipid levels, cigarette smoking status, and HbA1c values. Normal weight was defined as a BMI between 18.5 and 24.9 kg/m^2^, overweight as a BMI between 25 and 29.9 kg/m^2^, and obesity as a BMI of 30 kg/m^2^ or higher.^[18]^ Lipidemic subgroups were defined as follows: Group 1 included individuals with optimal lipid levels (TC < 200 mg/dL, LDL-C < 100 mg/dL, TG < 150 mg/dL); Group 2 included those with at least one borderline high lipid parameter (TC 200 to 239 mg/dL, LDL-C 100–129 mg/dL, TG 150–199 mg/dL); and Group 3 included those with at least 1 high lipid parameter (TC ≥ 240 mg/dL, LDL-C ≥ 130 mg/dL, TG > 200 mg/dL).^[5,19]^ An FG level of 100 to 125 mg/dL or HbA1c of 5.7% to 6.4% was considered indicative of prediabetes, while a FG level ≥ 126 mg/dL or HbA1c ≥ 6.5% was considered diagnostic for DM.^[20]^ Subsequently, each subgroup was compared internally in terms of biochemical parameters, MHR, and pulmonary function.

### 2.2. Statistical analysis

The study population was determined based on a priori power analysis conducted using G*Power 3.1 software (Faul, Erdfelder, Lang and Buchner; Heinrich Heine University, Düsseldorf, Germany, 2020). Drawing on previous literature,^[12,13]^ the analysis showed that, with the chosen sample size, the study has over 80% power to detect a medium effect size (*d* = 0.35) at a significance level of α = 0.05, ensuring reliable and meaningful comparisons between groups.

The characteristics of the subjects, including sex, age, BMI, and smoking status, as well as the results of FG, HbA1c, lipid profiles, MHR, and PFTs, were evaluated. There were no missing data. Continuous variables were presented as mean ± SD for normally distributed data and as median with interquartile range (1st–3rd quartiles) for non-normally distributed data, while categorical variables were expressed as counts and percentages. The normality of the data distribution was assessed using the Kolmogorov–Smirnov test. When the assumptions for parametric tests were satisfied, one-way ANOVA was performed to compare differences between independent groups. Otherwise, the Kruskal–Wallis test was employed to compare differences between independent groups. Differences were considered statistically significant at a 2-tailed *P*-value of <.05. Correlations between lipids and PFTs were evaluated using Spearman rank correlation coefficient (*r*), interpreted as follows: 0.00 to 0.25 = no or very weak correlation; 0.25 to 0.50 = weak correlation; 0.50 to 0.75 = moderate correlation; and 0.75 to 1.00 = strong correlation, with significance set at *P* < .05. Multivariable linear regression models were performed with spirometric parameters as dependent variables, while lipid levels and relevant covariates (age, BMI, and smoking status) were included as predictors. *F* values, *P* values, and effect sizes (η^2^) were calculated and reported. All data were analyzed using SPSS version 27.0 (IBM Corp., Chicago). The heatmap was created using Python (Python Software Foundation, version 3.10; matplotlib 3.7 and seaborn libraries 0.12).

## 3. Results

A total of 153 individuals participated in the study, with a balanced distribution of males and females (n = 78 and n = 75, respectively). The study population was predominantly middle-aged, with a median age of 44 years. One-third of participants were smokers, and the mean BMI indicated an overweight population. None of the participants reported alcohol use or occupational exposure, or had CVD or hypertension. All patients diagnosed with diabetes (n = 31, %20) had received their diagnosis within the last 10 years and were undergoing oral drug therapy. The characteristics, biochemical data, and PFT results of the participants are summarized in Table 1.The characteristics and biochemical test results of various subgroups classified by BMI, HbA1c levels, lipid levels, smoking status, and sex are presented in Table 2.

**Table 1:** Characteristics, biochemical parameters, and pulmonary function test results of all individuals.

	Results* (n = 153)
Age (yr)	44 (34.5–50)
Sex (n, %)	
Male	78 (51%)
Female	75 (49%)
BMI (kg/m^2^)	27.7 ± 4.6 (17.8–38.7)
Smoking	
Presence, n (%)	51 (33%)
Absence, n (%)	102 (67%)
Diabetes mellitus	
Presence, n (%)	31 (20%)
Absence, n (%)	122 (80%)
FG (mg/dL)	95 (86–106.2)
TC (mg/dL)	185.3 (160.5–206.2)
LDL-C (mg/dL)	108.3 ± 34.6 (28.3–265.3)
HDL-C (mg/dL)	45.7 (37.6–57.2)
TG (mg/dL)	128 (78.1–188.5)
HbA1c (%)	5.57 (5.28–6.06)
Monocyte count (×10^3^/μL)	0.44 (0.36–0.58)
MHR	0.009 (0.006–0.013)
FVC (L)	3.7 (3–4.5)
FVC (%)	97 (89–106)
FEV1 (L/s)	3.1 ± 0.8 (1.7–5.7)
FEV1 (%)	96 (88–104)
FEV1/FVC (%)	82.5 ± 6.9 (61.8–100)
FEV1/FVC ratio (%)	103 (98–109)
PEF (L/s)	6.23 ± 1.9 (1.9–3)
PEF (%)	80.1 ± 19.6 (45–133)

Continuous variables are presented as mean ± standard deviation for normally distributed data (*P* < .05) and as median (with interquartile range) for non-normal data (*P* > .05); categorical variables are shown as n (%).

BMI = body mass index, FEV1 = forced expiratory volume in 1 second, FG = fasting glucose, FVC = forced vital capacity, HbA1c = glycated hemoglobin, HDL-C = high-density lipoprotein cholesterol, LDL-C = low-density lipoprotein cholesterol, MHR = monocyte-to-HDL ratio, PEF = peak expiratory flow, TC = total cholesterol, TG = triglycerides.

* The distribution of all variables was assessed using the Kolmogorov–Smirnov test.

**Table 2:** Characteristics and biochemical test results of individuals classified by BMI, HbA1c levels, lipid levels, smoking status, and sex.

Group	Subgroup	Age (yr)	Sex (n, female/ male)	BMI (kg/m^2^)	Smoking (n, −/+)	FG (mg/dL)	TC (mg/dL)		LDL-C (mg/dL)		HDL-C (mg/dL)		TG (mg/dL)		HbA1c (%)†		Monocyte count (×10^3^/μL)		MHR
BMI (kg/m^2^)	1. Normal (n = 44)	37 ± 11	24/20	22 ± 2	28/16	88 (80–103)	170 ± 32		96 ± 30		54 ± 16		88 (60–128)		5.4 (5–5.7)		0.45 ± 0.14		0.009 ± 0.003
	2. Overweight (n = 55)	42 ± 10	24/31	27 ± 1	36/19	95 (87–106)	187 ± 36		107 ± 31		45 ± 13		158 (87–219)		5.5 (5.3–5.9)		0.49 ± 0.17		0.012 ± 0.006
	3. Obese (n = 54)	46 ± 7	27/27	32 ± 2	38/16	97 (88–107)	196 ± 42		118 ± 37		46 ± 18		138 (104–198)		5.7 (5.4–6.5)		0.46 ± 0.16		0.011 ± 0005
*P*-value		**.001** *	NS	**.001** *	NS	**.027** *	**.005** *	**.006** *		**.007** *		**.001** *		**.028** *		NS		NS	
HbA1c (%)	1. Normal (n = 88)	39 ± 9	43/45	26 ± 4	59/29	89 (84–98)	177 ± 35		104 ± 29		49 ± 14		103 (72–144)		5.3 (5.0–5.4)		0.46 ± 0.14		0.010 ± 0.005
	2.Prediabetes (n = 34)	43 ± 10	19/15	28 ± 4	21/13	96 (85–102)	202 ± 44		120 ± 42		51 ± 20		144 (95–179)		5.9 (5.7–6)		0.47 ± 0.19		0.010 ± 0.005
	3. DM (n = 31)	49 ± 8	13/18	29 ± 4	22/9	141 (106–242)	189 ± 36		106 ± 36		43 ± 14		198 (114–255)		6.9 (6.6–8.4)		0.49 ± 0.18		0.012 ± 0.006
*P*-value		**.001** *	NS	NS	NS	**.001** *	**.007** *	NS		NS		**.001** *		**.001** *		NS		NS	
Lipids (mg/dL)	1.Optimal (n = 40)	38 ± 10	23/17	25 ± 4	26/14	87 (80–100)	149 ± 23		76 ± 18		55 ± 21		77 (60–109)		5.3 (5–5.5)		0.41 ± 0.11		0.008 ± 0.003
	2. Borderline high (n = 54)	44 ± 10	26/28	27 ± 4	40/14	92 (85–103)	183 ± 19		109 ± 16		50 ± 13		117 (81–162)		5.6 (5.2–6)		0.46 ± 0.14		0.010 ± 0.005
	3. High (n = 59)	43 ± 9	26/33	29 ± 4	36/23	101 (92–126)	212 ± 40		128 ± 39		42 ± 13		204 (133–270)		5.8 (5.4–6.6)		0.51 ± 0.19		0.013 ± 0.006
*P*-value		**.015** *	NS	NS	NS	**.001** *	**.001** *	**.001** *		**.002** *		**.001** *		**.001** *		**.017** *		**.001** *	
Smoking	1. Absence (n = 102)	42 ± 9	62/40	27 ± 4	102/0	95 (86–106)	186 ± 40		107 ± 34		49 ± 17		124 (80–187)		5.5 (5.2–5.6)		0.44 ± 0.15		0.010 ± 0.005
	2. Presence (n = 51)	41 ± 11	13/38	27 ± 4	0/51	93 (81–105)	185 ± 38		110 ± 34		46 ± 13		130 (73–210)		5.6 (5.3–6)		0.52 ± 0.16		0.012 ± 0.006
*P*-value		NS	**.001** *	NS	**.001** *	NS	NS	NS		NS		NS		NS		**.005** *		**.006** *	
Sex	1. Male (n = 78)	42 ± 12	78/0	28 ± 4	40/38	97 (86–121)	181 (154–206)		104 ± 32		41 ± 14		175 ± 135		5.5 (5.2–6.2)		0.49 ± 0.16		0.013 ± 0.006
	2. Female (n = 75)	42 ± 8	0/75	27 ± 4	62/13	92 (85–101)	188 (168–212)		112 ± 36		56 ± 14		128 ± 70		5.6 (5.2–6)		0.44 ± 0.15		0.008 ± 0.004
*P*-value		NS	NS	NS	**.001** *	**.027** *	**.047** *	NS		**.001** *		**.027** *		NS		NS		**.001** *	

The distribution of all variables was assessed using the Kolmogorov–Smirnov test. Continuous variables are presented as mean ± standard deviation for normally distributed data (*P* < .05) and as median (with interquartile range) for non-normal data (*P* > .05).

BMI = body mass index, FG = fasting glucose, HbA1c = glycated hemoglobin, HDL-C = high-density lipoprotein cholesterol, LDL-C = low-density lipoprotein cholesterol, MHR = monocyte-to-HDL ratio, NS = nonsignificant, TC = total cholesterol, TG = triglycerides.

* *P* < .05 is considered significant.

† The NGSP (%HbA1c) and the IFCC (mmol/mol) relationship is: NGSP = (0.09148 × IFCC) + 2.152.

**Figure 1.**
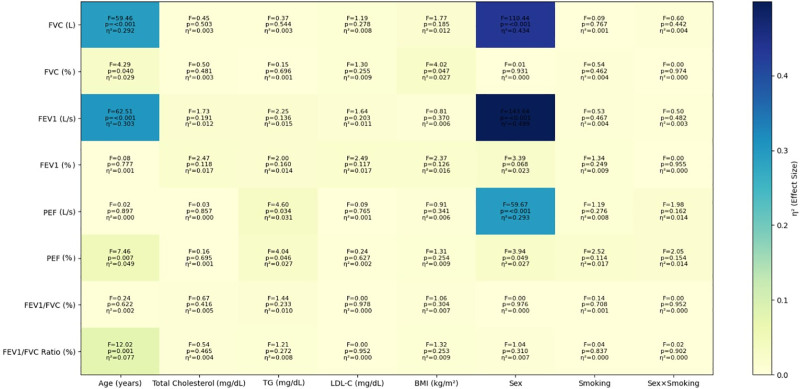
The associations of age, total cholesterol, TG, LDL-C, BMI, sex, smoking, and the sex × smoking interaction with pulmonary function tests. Each cell shows the *F*-value, *P*-value, and partial eta squared (η^2^, effect size). BMI = body mass index, FEV1 = forced expiratory volume in 1 second, FVC = forced vital capacity, LDL-C = low-density lipoprotein cholesterol, PEF = peak expiratory flow, TG = triglyceride.

PFT values, including FVC (L, %), FEV1 (L/s, %), FEV1/FVC (%), and PEF (L/s, %), are compared by subgroups in Table 3. Smokers had lower FVC and FEV1 values than nonsmokers (*P* = .008 and *P* = .005, respectively). Sex analysis revealed higher absolute FVC, FEV1, and PEF values in males. The associations of various predictors (age, cholesterol, TG, LDL-C, BMI, sex, smoking, and the sex × smoking interaction) with PFTs were analyzed using multivariable regression models, and the results, including *F* values, *P* values, and effect sizes, are presented as a heatmap in Figure 1. Increasing age was significantly associated with lower FVC, FEV1, PEF (%), and the FEV1/FVC ratio, with large effect sizes for FVC and FEV1. Sex demonstrated even stronger associations, showing very strong relationships with absolute lung volumes (FVC, FEV1, PEF), indicating marked differences between males and females. In contrast, lipid parameters generally showed weak or no associations, although TG levels were modestly related to PEF values. Similarly, BMI was only weakly associated with FVC (%). Neither smoking status nor its interaction with sex showed significant associations with respiratory outcomes. Overall, these findings highlight the dominant role of age and sex in pulmonary function, while metabolic factors such as lipids and BMI appear to play only minor roles.

**Table 3:** Pulmonary function test results classified by BMI, HbA1c levels, lipid levels, smoking status, and sex.

Group	Subgroup	FVC (L)	FVC (%)	FEV1 (L/s)	FEV1 (%)	FEV1/FVC (%)	FEV1/FVC ratio (%)	PEF (L/s)	PEF (%)
BMI (kg/m^2^)	1. Normal (n = 44)	3.97 ± 1.09	98 ± 13	3.26 ± 0.80	95 ± 12	82 ± 7	102 ± 8	6.0 ± 1.88	76 ± 19
	2.Overweight (n = 55)	3.95 ± 1.09	99 ± 13	3.25 ± 0.85	97 ± 12	82 ± 5	102 ± 7	6.3 ± 1.92	80 ± 17
	3. Obese (n = 54)	3.74 ± 1.05	99 ± 17	3.04 ± 0.78	97 ± 14	82 ± 7	103 ± 10	6.2 ± 1.94	82 ± 21
*P*-value		NS	NS	NS	NS	NS	NS	NS	NS
HbA1c (%)	1. Normal (n = 88)	3.93 ± 1.02	98 ± 14	3.24 ± 0.79	97 ± 13	83 ± 6	102 ± 8	6.3 ± 1.91	80 ± 19
	2.Prediabetes (n = 34)	3.95 ± 1.40	101 ± 17	3.20 ± 1.01	98 ± 14	81 ± 7	102 ± 10	6.2 ± 1.98	80 ± 18
	3. Diabetes (n = 31)	3.67 ± 0.77	97 ± 12	2.98 ± 0.61	96 ± 11	81 ± 6	103 ± 8	6.0 ± 1.88	79 ± 20
*P*-value		NS	NS	NS	NS	NS	NS	NS	NS
Lipids (mg/dL)	1. Optimal (n = 40)	3.99 ± 1.08	101 ± 14	3.25 ± 0.79	98 ± 15	82 ± 7	101 ± 9	6.3 ± 2.13	81 ± 21
	2. Borderline high (n = 54)	3.77 ± 1.07	97 ± 12	3.16 ± 0.88	97 ± 11	83 ± 5	104 ± 7	6.2 ± 2.00	80 ± 18
	3. High (n = 59)	3.91 ± 1.09	99 ± 17	3.16 ± 0.77	96 ± 12	81 ± 7	101 ± 9	6.1 ± 1.68	78 ± 19
*P*-value		NS	NS	NS	NS	NS	NS	NS	NS
Smoking	1. Presence (n = 51)	3.70 ± 0.99	99 ± 15	3.05 ± 0.77	97 ± 13	82 ± 6	102 ± 8	6.0 ± 1.73	81 ± 18
	2. Absence (n = 102)	4.23 ± 1.16	97 ± 12	3.44 ± 0.84	96 ± 12	82 ± 7	102 ± 9	6.6 ± 2.19	77 ± 21
*P*-value		**.008** *	NS	**.005** *	NS	NS	NS	NS	NS
Sex	1. Female (n = 75)	3.14 ± 0.59	99 ± 15	2.60 ± 0.43	95 ± 11	83 ± 6	102 ± 8	5.1 ± 1.16	78 ± 17
	2. Male (n = 78)	4.59 ± 0.95	98 ± 14	3.74 ± 0.69	98 ± 14	81 ± 6	102 ± 9	7.3 ± 1.89	81 ± 21
*P*-value		**.001** *	NS	**.001** *	NS	NS	NS	**.001** *	NS

The distribution of all variables was assessed using the Kolmogorov–Smirnov test. Continuous variables are presented as mean ± standard deviation for normally distributed data (*P* < .05).

BMI = body mass index, FEV1 = forced expiratory volume in 1 second, FVC = forced vital capacity, HbA1c = glycated hemoglobin, NS = nonsignificant, PEF = peak expiratory flow.

* *P* < .05 is considered significant.

## 4. Discussion

This study investigated the relationship between PFTs and metabolic parameters, including lipid levels, MHR, glucose, and HbA1c, in individuals undergoing routine health checkups. The findings revealed several notable associations, particularly regarding the relationships of age and sex with PFTs.

One of the main findings of this study is the lower FVC and FEV1 observed in smokers compared to nonsmokers; however, neither smoking status nor its interaction with sex was independently associated with respiratory outcomes in the multivariable analysis. Smoking is well-documented to impair lung function by promoting airway inflammation, oxidative stress, and airway remodeling.^[16,17]^ The impact of smoking on lung function is known to be strongly related to both the cumulative exposure and the duration of smoking, with higher intensity and longer duration leading to greater pulmonary impairment.^[21]^ Previous studies have also reported that smoking is an important risk factor for COPD and other lung diseases, often leading to reductions in PFT values^.[17,22]^ In the study, the lack of an independent association of smoking in the regression analysis may be due to the relatively low amount and short duration of smoking within the smoker group.

Sex differences in pulmonary function were also observed, with males exhibiting higher FVC, FEV1, and PEF compared to females. This finding is concordant with the general notion that males typically have greater lung volumes than females due to larger body size. The observed differences may be related to smoking habits, which were more prevalent in males (74% of the smokers) in this study. Additionally, hormonal differences and variations in muscle strength, body composition, and exposure to environmental risk factors might contribute to these sex-based discrepancies.^[16,17]^

The studies highlighted the relationship between age and lung capacity, indicating that lung function undergoes significant changes over the lifespan. The lung function typically peaks at the age of 25 and begins to decline more noticeably from around the age of 35. This decline is associated with natural aging processes, including weakening of respiratory muscles, loss of lung tissue elasticity, and changes in the rib cage structure. These findings underscore the importance of considering age when assessing lung health and highlight the need for early interventions to maintain optimal lung function throughout life.^[23,24]^

While age and sex showed significant associations with lung function, the relationships of lipid levels, BMI, and HbA1c with PFTs were relatively weak. Previous studies have suggested that obesity and dyslipidemia might negatively affect lung function through mechanisms like systemic inflammation and altered lipid metabolism in lung tissues.^[7–9]^ However, in this study, BMI, HbA1c, and lipid levels did not show significant associations with FVC, FEV1, or other PFT parameters. This lack of association might be due to the relatively healthy status of the study population, the exclusion of individuals with chronic lung diseases, or the fact that changes in lipid metabolism may primarily affect lung function in individuals with more severe metabolic dysfunctions, such as those with diabetes or CVDs.^[4,12,13]^ Additionally, all patients with diabetes had received their diagnosis within the last 10 years and were undergoing treatment, which may explain why diabetes did not appear to affect lung function in this study.^[25]^ The study’s results may also have been influenced by the small sample size.

The analysis shows significant biochemical and clinical differences between males and females, particularly in lipid profiles, FG levels, and MHR. These differences may imply that males could be at a higher risk for CVDs and metabolic disorders and need further exploration.^[15,26–29]^ The results related to MHR and monocyte count were also remarkable. Higher MHR levels in smokers and males may indicate heightened systemic inflammation and oxidative stress in these groups. Similarly, the study reported that elevated MHR is associated with cigarette smoking and can be used as an indicator of a systemic inflammatory response in smokers.^[26]^ A similar study underlined that sex (male), high BMI, and metabolic syndrome increase MHR in the subjects.^[27]^ MHR calculated during routine complete blood count and biochemical tests can easily identify healthcare workers for the prevention and early detection of lifestyle-related diseases. This study suggests further use of MHR in routine screening. Additionally, MHR has been proposed as a marker of inflammation and is linked to various cardiovascular and metabolic disorders.^[15,28,29]^ In our study, the high lipid groups had a higher MHR similar to the previous studies.^[15,28–30]^ Endothelial dysfunction is a precursor to atherosclerosis and is strongly associated with the progression of cardiovascular and microvascular complications. The researchers underlined the possibility that MHR could be used not only as a marker of inflammation but also as an indicator of endothelial dysfunction in patients with T2DM. Early detection of endothelial dysfunction using a marker like MHR could allow for more targeted interventions to prevent atherosclerosis and its complications.^[28–30]^ Although we found a higher MHR rate in diabetic patients compared to others, there was no statistical difference in MHR between the groups. This may be because diabetic patients who underwent checkups have not had this disease for a long time or have been on controlled treatment. Also, these patients did not report any micro- or macrovascular complications with DM. Indirectly, the lack of significant differences in PFTs across diabetic subgroups suggests that while MHR may reflect systemic inflammation, it may not directly translate into pulmonary function impairment in this population.

This study emphasizes that both metabolic factors (lipids, HbA1c) and behavioral factors (obesity, smoking, alcohol, occupational exposure) are important in assessing pulmonary function. MHR may serve as a potential clinical marker for inflammation and oxidative stress. The sex differences observed in PFTs highlight the need for further research to explore how sex-specific factors are related to lung health. Although lipid levels, BMI, and HbA1c did not show strong associations with PFTs in this study, their role in pulmonary diseases, particularly in populations with more severe metabolic disturbances, needs further investigation.

This study has certain limitations. The sample sizes of the subgroups were relatively small. Details regarding the specific treatment regimens of patients with diabetes and HL were not available. Information on participants’ physical activity levels, as well as the amount and duration of smoking, was also not available. Additionally, the exclusion of individuals with chronic diseases may have limited the generalizability of the findings to healthier populations. Due to the cross-sectional design of this study, causal relationships between PFTs and metabolic parameters cannot be established; therefore, our findings should be interpreted as associations rather than evidence of causality. Future longitudinal studies with larger and more diverse populations may provide further insights into the complex relationships between lipid metabolism, systemic inflammation, and lung function.

In conclusion, age and sex are significantly associated with pulmonary function, while the associations of smoking, BMI, HbA1c, and lipid levels appear to be more complex and require further investigation. Efforts to reduce smoking and manage metabolic health remain crucial for preserving lung function and preventing respiratory diseases. However, associations with nonmodifiable factors such as age and sex cannot be altered; they remain important determinants of lung function.

## Acknowledgments

The authors gratefully acknowledge Ramazan Aksoy, Ayşegül Dane, Kezban Yaman, and Havali Akkaya for their technical support during this study.

## Author contributions

**Conceptualization:** Kadriye Akpinar.

**Investigation:** Kadriye Akpinar, Alev Lazoğlu Özkaya.

**Methodology:** Kadriye Akpinar, Alev Lazoğlu Özkaya.

**Writing – original draft:** Kadriye Akpinar, Alev Lazoğlu Özkaya.
